# Pegylated Interferon α-2a Triggers NK-Cell Functionality and Specific T-Cell Responses in Patients with Chronic HBV Infection without HBsAg Seroconversion

**DOI:** 10.1371/journal.pone.0158297

**Published:** 2016-06-27

**Authors:** Juliana Bruder Costa, Tania Dufeu-Duchesne, Vincent Leroy, Inga Bertucci, Magali Bouvier-Alias, Noelle Pouget, Ophelie Brevot-Lutton, Marc Bourliere, Fabien Zoulim, Joel Plumas, Caroline Aspord, ANRS HB06 PEGAN study group

**Affiliations:** 1 University Grenoble Alpes, Grenoble, F-38041 France; INSERM, U1209, Immunobiology and Immunotherapy of Chronic Deseases, La Tronche, F-38706 France; 2 CHU Grenoble, Michallon Hospital, Hepato-gastroenterology unit, Grenoble, F-38043 France; 3 University Grenoble Alpes, Grenoble, F-38041 France; INSERM, U1209, Analytic Immunology of chronic pathologies, La Tronche, F-38706 France; 4 ANRS (France REcherche Nord & sud Sida-hiv Hépatites: FRENSH), Paris, France; 5 Department of Virology, Henri Mondor Hospital, University Paris-Est and Inserm U955, Creteil, France; 6 Sorbonne Universités, UPMC Univ Paris 06, INSERM, Institut Pierre Louis d’épidémiologie et de Santé Publique (IPLESP UMRS 1136), 75012, Paris, France; 7 Hepato-gastroenterology department Hospital Saint Joseph, Marseille, 13008 France; 8 INSERM U1052—CNRS 5286, Cancer Research Center of Lyon (CRCL), Lyon, France; 9 Hepatology Department, Hospices Civils de Lyon, Lyon, France; 10 Université de Lyon, Lyon, France; 11 EFS Rhone-Alpes, R&D Laboratory, La Tronche, F-38701 France; CRCL-INSERM, FRANCE

## Abstract

Pegylated interferon α-2a (Peg-IFN-α) represents a therapeutic alternative to the prolonged use of nucleos(t)ide analog (NA) in chronic hepatitis B (CHB) infection. The mechanisms leading to a positive clinical outcome remain unclear. As immune responses are critical for virus control, we investigated the effects of Peg-IFN-α on both innate and adaptive immunity, and related it to the clinical evolution. The phenotypic and functional features of the dendritic cells (DCs), natural killer (NK) cells and HBV-specific CD4/CD8 T cells were analyzed in HBeAg-negative CHB patients treated for 48-weeks with NA alone or together with Peg-IFN-α, before, during and up to 2-years after therapy. Peg-IFN-α induced an early activation of DCs, a potent expansion of the CD56^bright^ NK subset, and enhanced the activation and functionality of the CD56^dim^ NK subset. Peg-IFN-α triggered an increase in the frequencies of Th1- and Th17-oriented HBV-specific CD4/CD8 T cells. Peg-IFN-α reversed the unresponsiveness of patients to a specific stimulation. Most of the parameters returned to baseline after the stop of Peg-IFN-α therapy. Peg-IFN-α impacts both innate and adaptive immunity, overcoming dysfunctional immune responses in CHB patients. These modulations were not associated with seroconversion, which questioned the benefit of the add-on Peg-IFN-α treatment.

## Introduction

Pegylated interferon α-2a (Peg-IFN-α) therapy represents a promising therapeutic alternative to the prolonged use of nucleos(t)ide analogs (NA) in chronic hepatitis B (CHB) infection [1–4]. Although Peg-IFN-α potentially leads to HBsAg seroconversion, its mechanisms of immunomodulation remain poorly known.

HBV modulates innate and adaptive immunity to escape clearance, generating weak and dysfunctional immune responses. Dysfunctions in dendritic cells (DCs), natural killer (NK) cells and T cells have been identified in patients with CHB infection. The virus may actively alter the function of plasmacytoid DCs (pDCs) [5], leading to a failure of the subsequent pDC-NK cross-talk in CHB patients [6]. Defects in the activation and antiviral functions of NK cells have also been described [7]. In addition, HBV-specific T-cell responses are often weak in patients who evolve toward chronic HBV infection [8], whereas multi-specific and vigorous HBV-specific T-cell responses directed toward epitopes located within the major HBV proteins [i.e. the nucleoscapsid (HBc), the surface antigen (HBs), the HBx antigen, and the polymerase (POL)] are required to successfully control HBV infection [9].

Peg-IFN-α represents a promising way to boost innate and adaptive immunity to overcome dysfunctional immune responses. IFN-α is a pleiotropic cytokine that displays strong antiviral and immunomodulatory properties [10]. It is produced in large amounts by pDCs during the early stages of viral infection. IFN-α can directly inhibit viral replication and enhance antiviral responses by acting on different immune effectors such as NK and T cells [10, 11]. NK cells play a pivotal role in antiviral immunity by controlling viral replication through direct cytotoxicity or by the production of immunoregulatory cytokines including IFN-γ and TNF-α that can modulate adaptive immune responses [12][13]. Virus-specific T cells are crucial in the later stages of viral infection. Following their activation by innate effectors, such as DCs and activated NK cells, virus-specific CD8+ T lymphocytes and CD4+ T-helper cells can control the infection through the secretion of pro-inflammatory cytokines and by differentiation into cytotoxic effectors that can lyse the infected cells [14].

The clinical benefit of Peg-IFN-α (as mono- or combination therapy) is superior to NA alone, whereas there is no difference in the virological response between treatment with Peg-IFN-α as monotherapy or in combination with NA [3, 4, 15, 16]. The precise impact of this therapy on the key antiviral effectors and the mechanism leading to a positive clinical outcome remain not fully understood. Only one study compared immunological changes triggered by Peg-IFNα alone, NA alone or the combination of both, but on limited immune parameters and at very early time points (within the first two weeks of therapy) [17]. Peg-IFN-α as a monotherapy activates DCs [18], expands and modulates the function of CD56^bright^ NK cells [19, 20], and drives either an improvement or no changes in HBV-specific T-cell responses [21–23]. These studies were performed in separate cohorts of patients, thus preventing correlations between the immune parameters. As well, the kinetics of the immunologic changes was not detailed, which prevented the distinction of early and late effects. Finally, the studies did not feature long-term follow-up after the cessation of the treatment or comparison of the combined therapy with NA alone.

To overcome these limitations, the current sub-study investigated the impact of Peg-IFN-α on all major antiviral immune effectors including pDCs, mDCs, CD56^bright^CD16^+/-^ and CD56^dim^CD16^+/-^ NK-cell subsets, and T cells in the same cohort of HBeAg-negative CHB patients. We compared patients receiving a 48-week course of Peg-IFN-α in addition to NA with patients treated with NA alone. The immune parameters were studied at baseline, at different time points during the Peg-IFN-α therapy, and up to 2 years after the end of the treatment. We provide a dynamic longitudinal analysis of the features of both innate and adaptive responses, and related this to changes in the clinical parameters. This work contributes to a better understanding of the impact of Peg-IFN-α on immunity, revealing yet unexplored immune effects of the combination therapy. The observed alterations being not prognostically relevant, our work also questioned the benefit of the add-on Peg-IFN-α treatment over the NA or Peg-IFN-α monotherapies.

## Materials and Methods

### Patients

The study participants comprised 23 HBsAg-positive and HBeAg-negative CHB patients treated by analogs who had undetectable HBV-DNA for at least one year and who were enrolled in a multicenter, randomized, phase 3 study of Peg-IFN-α (ANRS HB06-PEGAN: NCT01172392). Fourteen patients remained on NA alone (control group) and nine received an additional 180μg of Peg-IFN-α (Pegasys; F Hoffmann-La Roche) once a week for 48 weeks (Peg-IFN-α group) (Table 1). Seven out of 14 patients in the control group and 7 out of 9 patients in the Peg-IFN-α group were HLA-A*02:01+ (Table 1). The study protocol was conducted according to the Declaration of Helsinki and French law for biomedical research, and approved by the Ethics Committee “Comite de Protection des Personnnes” (CPP) Sud-Méditerranée-I and the French Regulatory Authority “Agence Nationale de Securite du Medicament et des produits de sante” (ANSM). All participants gave the written informed consent. Heparinized blood samples were obtained at baseline and after 4 (Peg-IFN-α only), 12, 24, 48, 96 and 144 weeks of treatment. Peripheral blood mononuclear cells (PBMCs) were purified by Ficoll-Hypaque density gradient centrifugation (Eurobio) and the total lymphocyte concentration was determined. All the experiments were performed with freshly purified PBMCs.

**Table 1:** Clinical features of patients at baseline and during the course of the treatment.

					Baseline					HBsAg (log IU/ml)						Anti-HBs Ab status			
Case #	Group	Age (year)	Sex	Analog	HBV DNA (IU/ml)	AST (IU/ml)	ALT (IU/ml)	Metavir activity	Metavir fibrosis	W0	W12	W24	W48	W96	W144	W0	W48	W96	W144
1^b^	**NA**	55	M	tenofovir	<20	22	21	2	4	2,85	2,97	2,93	2,89	2,83	ND	neg	neg	neg	neg
2	** **	54	M	entecavir	<20	27	24	1	1	3,17	3,15	3,16	3,02	2,82	ND	neg	neg	neg	neg
3	** **	56	M	tenofovir	<20	38	54	ND	4	1,72	1,72	1,72	1,65	1,60	ND	neg	neg	neg	neg
4^b^	** **	34	M	entecavir	<20	27	51	1	0	3,71	3,74	3,51	3,54	3,49	ND	neg	ND	neg	ND
5^b^	** **	31	M	tenofovir	<20	28	28	1	0	4,07	3,97	3,99	4,13	3,98	ND	neg	neg	neg	ND
6	** **	51	M	tenofovir	<20	22	35	ND	ND	3,08	2,99	3,04	3,05	3,05	2,80	neg	ND	neg	neg
7^b^	** **	39	M	entecavir	<20	19	51	3	3	3,11	3,08	3,13	3,11	3,09	2,92	neg	neg	neg	neg
8	** **	54	M	tenofovir	<20	23	29	2	2	3,41	3,32	3,34	3,16	3,08	ND	neg	ND	neg	neg
9	** **	35	M	tenofovir	<20	35	38	2	4	3,39	3,28	3,36	3,19	3,12	ND	neg	neg	neg	ND
10^b^	** **	48	F	lamivudine	<20	19	15	3	1	2,60	2,55	2,38	2,56	2,51	2,24	neg	neg	neg	neg
11	** **	65	M	tenofovir	<20	30	25	1	4	0,61	0,61	0,18	0,18	-0,52	ND	neg	neg	neg	pos
12	** **	37	M	entecavir	<20	43	35	2	1	4,32	4,30	4,21	4,14	4,42	ND	neg	neg	neg	ND
13^b^	** **	62	F	lamivudine	<20	23	19	1	2	3,36	ND	3,40	3,22	3,35	ND	neg	neg	neg	neg
14^b^	** **	35	M	tenofovir	<20	24	24	2	4	3,51	3,44	3,50	3,57	3,47	3,54	neg	neg	neg	ND
15^b^	**NA + Peg-IFNα**	41	M	entecavir	<20	28	39	3	2	3,66	3,55	3,53	3,00	3,53	3,37	neg	neg	neg	neg
16^b^	** **	44	M	entecavir	<20	23	41	1	2	3,35	3,21	3,24	3,10	3,36	ND	neg	neg	neg	neg
17^b^	** **	63	M	tenofovir	<20	31	42	1	1	2,93	2,97	2,83	2,59	2,65	ND	neg	neg	neg	ND
18	** **	57	M	entecavir	<20	19	28	2	4	2,88	2,89	2,79	2,67	2,61	2,34	neg	neg	neg	neg
19^b^	** **	49	M	entecavir	<20	32	20	ND	ND	3,90	3,78	2,70	1,28	3,26	2,90	neg	neg	neg	neg
20^a^,^b^	** **	49	F	tenofovir	<20	17	13	1	2	2,38	2,24	2,36	2,20	1,61	ND	neg	neg	neg	ND
21^b^	** **	67	M	tenofovir	<20	40	56	2	2	3,78	3,64	3,24	1,83	3,08	3,06	neg	neg	neg	neg
22	** **	34	M	entecavir	<20	24	22	ND	ND	4,36	4,27	4,27	4,34	4,35	ND	neg	neg	neg	ND
23^b^	** **	61	M	adefovir	<20	25	25	2	4	2,89	2,84	2,31	2,21	1,86	ND	neg	neg	ND	ND

a: patient stopped treatment after W12

b: patients HLA-A*02:01+

NA: nucleos(t)ide analog

ND: not determined.

### Phenotypic analysis

PBMCs were stained with fluorochrome-labeled anti-human antibodies or isotype-matched controls. T cells were defined with CD3/CD4/CD8/CD25 (BD), and Foxp3 (eBioscience) antibodies (Abs), and NK subsets were defined using CD3, CD16, and CD56 Abs (BD). The activation status was evaluated using the CD69 marker (BD). pDCs and mDCs were defined with BDCA2 (Miltenyi) and HLA-DR,/CD11c/Lin Abs (BD), and their activation status using CD40/CD80/CD86 Abs (BD). The stained cells were analyzed by flow cytometry using a FACSCantoII device equipped with Diva software (BD). Absolute numbers (cells/ml blood) were obtained by multiplying their percent by the total lymphocyte number. To ensure quality control during the study, we performed a systematic standardization of the fluorescence intensities using cytometer setup and tracking beads (BD).

### NK function measurements

Cultures were performed in RPMI1640-Glutamax supplemented with 1% non-essential amino acids, 100μg/mL gentamycin, 10% fetal calf serum (Invitrogen) and 1mM sodium pyruvate (SigmaAldrich). PBMCs were cultured in the presence or absence of recombinant human interleukin (rhIL)12 and rhIL18 (R&D Systems) for 18h. To measure IFN-γ secretion, 1μl/ml brefeldinA (BD) was added for the last 4h. Cells were labeled with CD3, CD16, and CD56 Abs (BD) and stained for intracellular IFN-γ (BD). NK cytotoxic activity was evaluated by a CD107 degranulation assay. The cultures were washed and co-cultured with K562 cells (5:1 ratio) for 3h. Anti-human CD107a/b Abs (BD) were added at the start of the re-stimulation together with GolgiSTOP for the last 2h. The cells were labeled with CD3, CD16, and CD56 Abs before flow cytometry analysis.

### Evaluation of HBV-specific T-cell responses

PBMCs were stimulated during 14 days without or with pools of 15-mer peptides overlapping by 10-residues covering the overall sequence of HBc, HBs, HBx, and POL antigens of HBV genotype D (pooled in 14 mixtures). The cultures were re-stimulated at day 7 with the same peptide pools, IL-2 (Proleukine) and 30Gy-irradiated allogeneic PBMCs. To determine the frequency of specific T cells, intracellular cytokine staining was performed at day 14 upon specific stimulation. Expanded T-cell lines were incubated for 5h with the corresponding peptide pools in the presence of brefeldinA for the last 4h. The cells were washed and stained with CD3/CD8 Abs (BD) followed by the intracellular staining of TNF-α, IFN-γ and IL-10. To determine the global cytokine profile, expanded T-cell lines were resuspended at 1x10^6^cells/ml, and restimulated with the corresponding peptide pools before measurement of cytokine secretion in the supernatant 24h later by a cytometric bead array (CBA) assay (BD).

### Measurements of specific CD8 T cells using tetramers

For HLA-A*02:01+ patients, specific T cells were measured directly from PBMCs by tetramer labeling using HBc18-27 (FLPSDFFPSV) and HBs335-343 (WLSLLVPFV) HLA-A*02:01-tetramers (Beckman Immunomics) followed by staining with CD3/CD8 Abs (BD).

### Induction of HBV-specific CD8 T-cell responses using pDC-based stimulation

The ability of HLA-A*02:01+ patients to respond to a stimulation by peptide-loaded HLA-A*02:01+ pDCs was evaluated as previously described [24]. PBMCs were co-cultured for 14 days with HBc18-27 and HBs335-343 peptide-loaded pDCs (1:10 ratio) and re-stimulated weekly in the presence of 200UI/ml IL-2 (Proleukine, Chiron). Specific T-cell responses were measured by tetramer labeling as previously described.

### Cytokine quantification

Plasma samples were collected before and at each time point of treatment, and the levels of IP-10 were quantified by a CBA assay (BD).

### Statistical analyses

Statistical analysis was performed using the Mann-Whitney non-parametric U-test, Wilcoxon matched pairs test, and Spearman correlation using Prism software (GraphPad Software).

## Results

### Peg-IFN-α increases the basal activation levels of DC subsets

**Fig 1:**
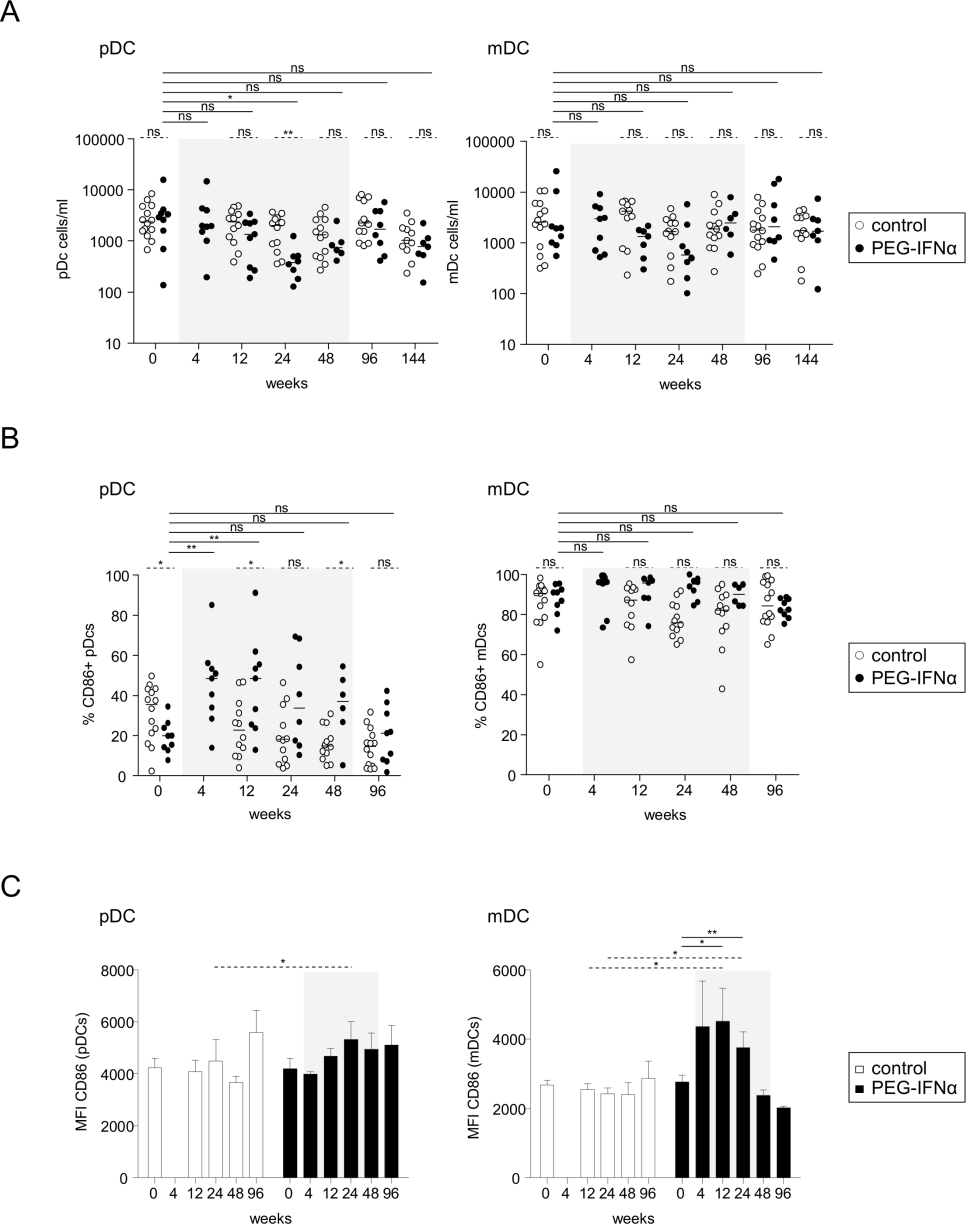
Modulation of DC subsets by Peg-IFN-α. Patients with CHB infection were treated with NA alone (*open circles*, n = 11–14) or together with Peg-IFN-α (*black circles*, n = 7–9). DC subsets were analyzed by flow cytometry before and at different time points of treatment. (A) Absolute numbers of pDCs and mDCs. (B) Basal percentages of CD86 expression on pDCs and mDCs. (C) Mean fluorescence intensity of CD86 on pDCs and mDCs. CD86 was not evaluable for W144. The gray area represents the period of Peg-IFN-α administration. Bars represent median. *P*-values were calculated using the Wilcoxon test (*straight lines*) or the Mann-Whitney test (*dashed lines*). *p<0.05, **p<0.01, ***p<0.001.

We first investigated the impact of Peg-IFN-α on the features of DC subsets. The frequency, absolute numbers, and activation status of pDCs (HLA-DR^+^BDCA2^+^) and mDCs (Lin-HLA-DR^+^CD11c^+^) were analyzed in both treatment arms before and at different times during treatment. We observed a continuous trend of a decrease in the frequencies and absolute numbers of DCs, achieving significance for pDCs at week (W) 24 (Fig 1A). Peg-IFN-α induced an up-regulation of CD86 expression on pDCs and mDCs (Fig 1B and 1C) without clearly affecting CD40 levels (S1 Fig). All the parameters returned to baseline after the cessation of the treatment. Despite the reduction of DC numbers over time, the activation status of pDCs and mDCs was modulated as soon as 4 weeks by Peg-IFN-α therapy.

### Peg-IFN-α modulates NK-cell distribution and function

**Fig 2:**
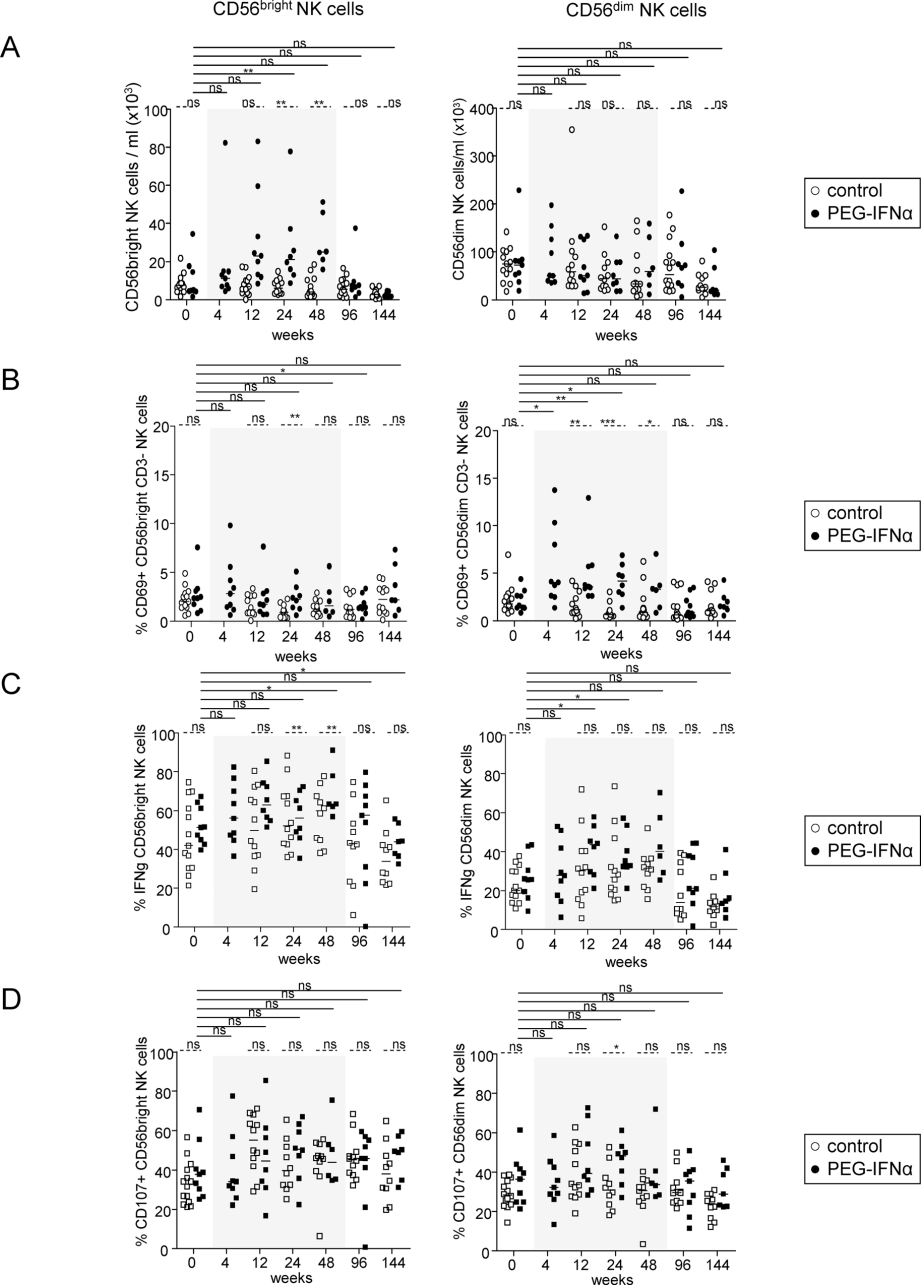
Modulation of NK-cell features by Peg-IFN-α. Patients with CHB infection were treated with NA alone (*open circles*, n = 12–13) or together with Peg-IFN-α (*black circles*, n = 8–9). CD56^+^CD3^-^ total NK cells and CD56^bright/dim^ NK subsets were analyzed by flow cytometry before and during Peg-IFN-α treatment. (A) Absolute numbers of CD56^bright^ and CD56^dim^ NK cells. (B) Basal level of CD69 expression. (C) IFN-γ secretion was evaluated by intracellular staining upon IL12/IL18 stimulation. (D) The cytotoxic activity was evaluated upon IL12/IL18 stimulation after co-culture with K562 by measuring CD107 surface expression. Bars represent median. *P*-values were calculated using the Wilcoxon test (*straight lines*) or the Mann-Whitney test (*dashed lines*). *p<0.05, **p<0.01, ***p<0.001.

We next assessed the impact of Peg-IFN-α on NK-cell subsets. Peg-IFN-α drove an increase in total NK-cell frequency (S2A Fig), attributable to a continuous enhancement of the CD56^bright^ NK-cell subset (Fig 2A), including both CD16^+^ and CD16^-^ CD56^bright^ NK cells (S2B Fig). NK cells were activated by Peg-IFN-α as soon as 4 weeks, as revealed by the increase in CD69 expression W4 post-treatment (S3A Fig). This modulation was seen only within the CD16^+^ and CD16^-^ CD56^dim^ NK cells (Fig 2B, S3B Fig). A significant increase of the ability of NK cells to secrete IFN-γ following IL12/IL18 stimulation in the Peg-IFN-α group was evident from W12 to W48 post-treatment compared to baseline and to the control group (Fig 2C, S4A and S4B Fig). In addition, the cytotoxic activity of NK cells assessed by CD107 surface expression upon IL12/IL18 stimulation and co-culture with the NK-cell target K562 was significantly improved in patients treated with Peg-IFN-α, peaking at W24 post-treatment (Fig 2D, S4C and S4D Fig). Thus, Peg-IFN-α drastically modulated NK-cell distribution and activation, and potentiated the functionality of the CD56^bright^ and CD56^dim^ NK-cell subsets. All the parameters returned to baseline after the cessation of treatment.

### Peg-IFN-α triggers Th1- and Th17-oriented HBV-specific CD4 and CD8 T-cell responses

We then investigated the immunomodulatory effects of Peg-IFN-α on the T-cell subsets. A gradual decrease in the absolute numbers of CD4/CD8 T cells was evident at W24 and W48 following Peg-IFN-α treatment accompanied by a slight modulation of their activation status (S5A and S5B Fig). Peg-IFN-α also induced a transient and modest modulation of the absolute numbers of regulatory T cells (Treg) (S5C Fig).

**Fig 3:**
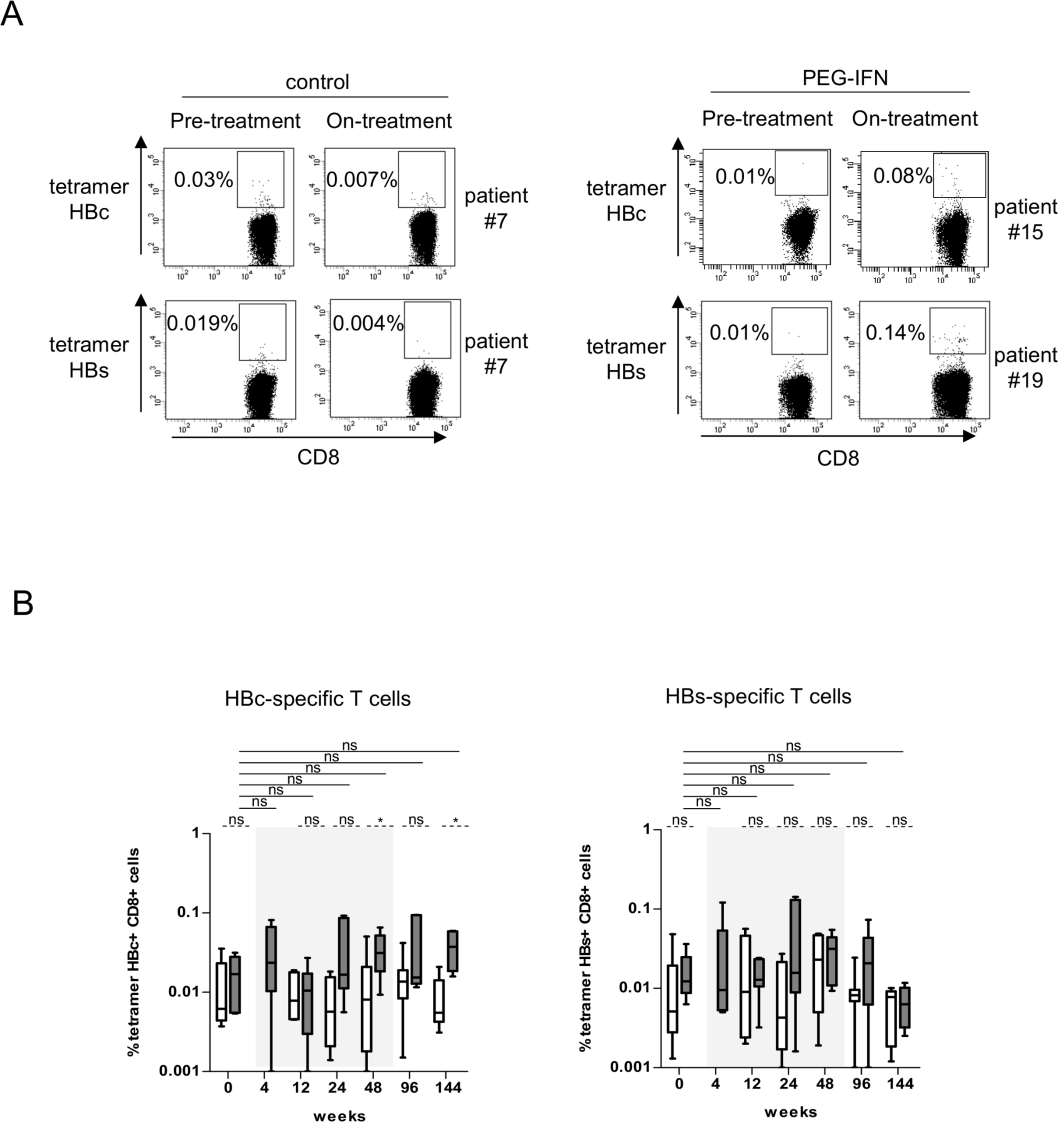
Direct HBV-specific CD8 T-cell response evolution during Peg-IFN-α therapy. Direct HBV-specific CD8 T cells measurements following Peg-IFN-α treatment using tetramers. (A) Representative dot plots of the tetramer labeling of HBc_18-27_- and HBs_335-343_-specific T cells (gated on CD8 T cells) before and 24 weeks after treatment in patients treated with nucleos(t)ide analog alone (*left panel*) or together with Peg-IFN-α (*right panel*). (B) Evolution of the basal percentages of HBc_18-27_- (left panel) and HBs_335-343_- (right panel) specific CD8 T cells in patients with CHB infection treated with nucleos(t)ide analog alone (*white bars*, n = 7) or together with Peg-IFN-α (*grey bars*, n = 7). The gray area represents the period of Peg-IFN-α administration.

**Fig 4:**
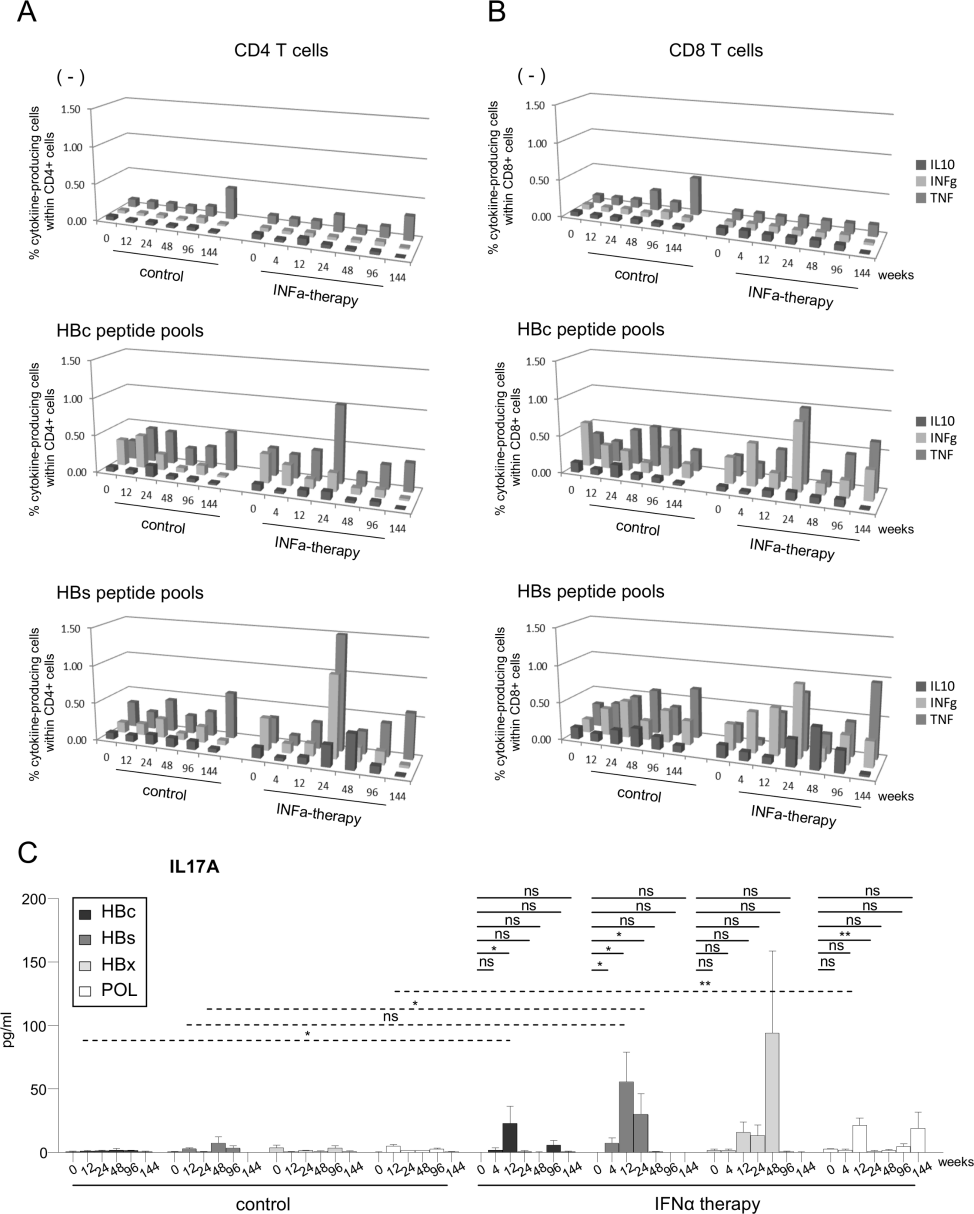
Frequencies and Th1/Th17 orientation of HBV-specific CD4/CD8 T cells during the course of Peg-IFN-α treatment. (A-B) Frequencies of HBV-specific CD4/CD8 T cells were evaluated before and during Peg-IFN-α treatment upon the stimulation of PBMCs with peptide pools and intracellular TNF-α, IFN-γ and IL-10 cytokine staining from patients treated with NA alone (n = 5–8) or together with Peg-IFN-α (n = 3–5). (A) Frequencies of cytokine-producing HBc-/HBs-specific CD4 T-cell responses (mean values). (B) Frequencies of cytokine-producing HBc-/HBs-specific CD8 T-cell responses (mean values). (C) IL-17A production was analyzed in supernatants of PBMCs stimulated with peptide pools derived from the HBc, HBs, HBx and POL antigens before and during Peg-IFN-α treatment in patients with CHB infection treated with NA alone (n = 10) or together with Peg-IFN-α (n = 9). *P*-values were calculated using the Wilcoxon test (*straight lines*) or the Mann-Whitney test (*dashed lines*). *p<0.05, **p<0.01, ***p<0.001.

To assess whether Peg-IFN-α therapy could improve the virus-specific T-cell responses, we first evaluated the ex-vivo frequency of HBc_18-27_ and HBs_335-343_-specific CD8 T cells directly from the blood of HLA-A*02:01+ patients using multimers. Notably, the frequency of circulating HBV-specific CD8 T cells following Peg-IFN-α therapy increased in some patients (Fig 3). We further analyzed the frequency and Th orientation of HBV-specific CD4/CD8 T cells following stimulation with overlapping peptide pools derived from HBc, HBs, HBx, and POL antigens. Upon specific stimulation, we observed the improvement of TNF-α and/or IFN-γ producing HBc- and HBs-specific T cells among CD4 (Fig 4A) and CD8 (Fig 4B) T cells during the course of Peg-IFN-α therapy compared to the control group and to the control stimulation. No elicitation of HBx- and pol-specific immune responses were noticed (S6 Fig). To obtain a more extended view of the Th profile of the activated T cells, we also measured the global cytokine profile in the culture supernatants following the specific stimulation of PBMCs with HBV-derived peptide pools. Strikingly, we observed a substantial secretion of IL-17A in response to the HBV antigens in patients treated with Peg-IFN-α compared to NA alone (Fig 4C). Nine out of 9 patients from the Peg-IFN-α group displayed such a Th17 profile at least at one time point, in response to 1 to 3 HBV antigens. Thus, Peg-IFN-α triggered the immune system toward Th1-biased HBV-specific T-cell responses and Th17 profile.

### Peg-IFN-α treatment reverses unresponsiveness to specific stimulation by HBV-derived peptide-loaded pDCs

**Fig 5:**
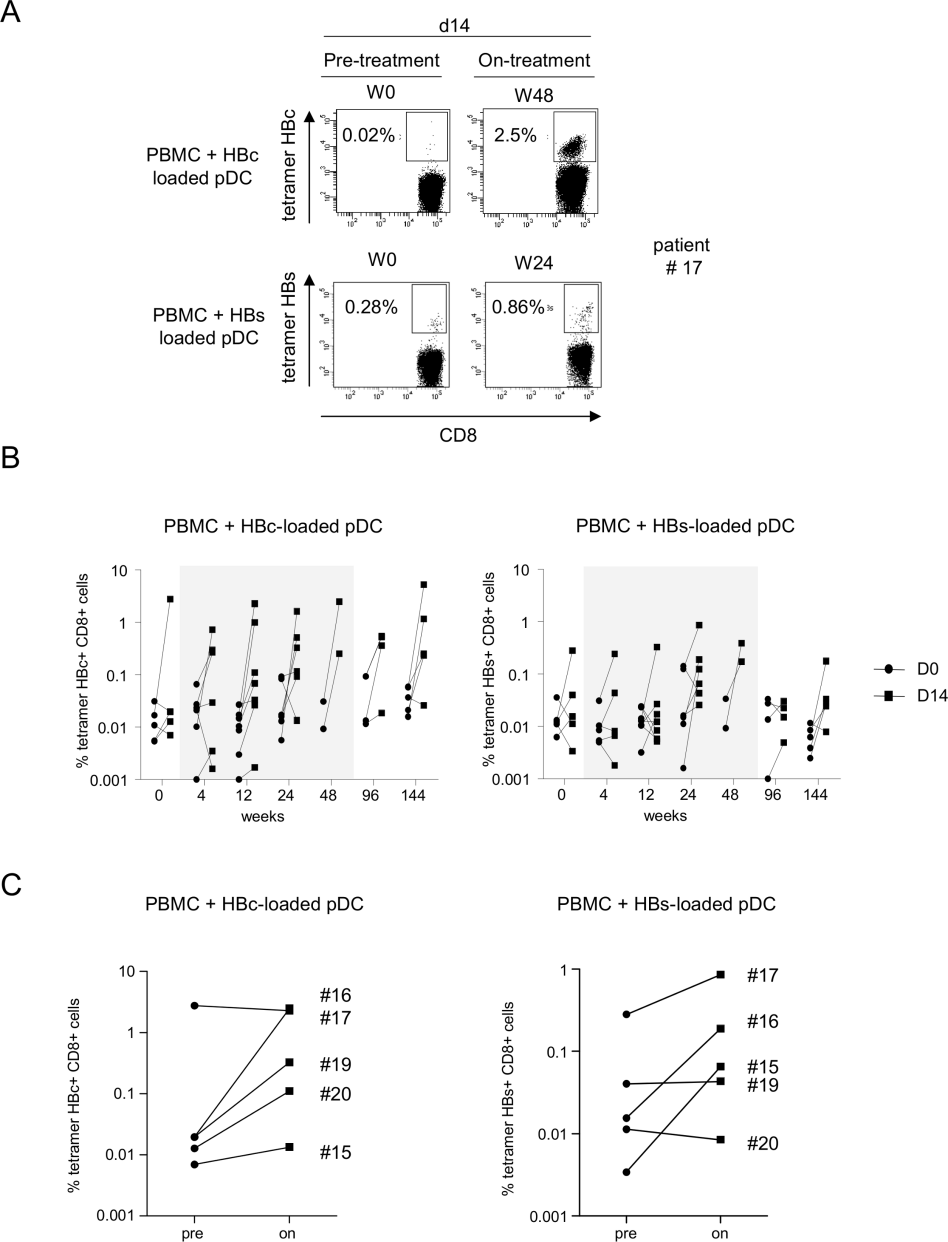
Peg-IFN-α reverses the unresponsiveness of patients to the stimulation by HBV-derived peptide-loaded pDCs. PBMCs were stimulated with pDCs loaded with HLA-A*02:01-restricted HBc_18-27_ and HBs_335-343_ peptides and the amplification of HBV-specific T cells was assessed using HLA-A*02:01-tetramers. (A) Representative dotplots of the tetramer labelling of HBc_18-27_- (*upper panel*) and HBs_335-343_- (*lower panel*) specific T cells (gated on CD8 T cells) at day (D)14 before and on Peg-IFN-α therapy. (B) Evolution of the percentages of HBc_18-27_- and HBs_335-343_-specific T cells at D0 and D14 of culture during Peg-IFN-α therapy. (C) Comparative levels of HBc_18-27_- and HBs_335-343_-specific CD8 T cells at D14 between baseline and on-treatment (n = 5). The gray area represents the period of Peg-IFN-α administration.

We previously developed a powerful technology based on a human HLA-A*02:01+ pDC line to trigger functional HBV-specific T-cell responses *ex-vivo* from chronic HBV patients [24]. We identified responder and non-responder patients, allowing the defining of an immunologic responsiveness to this strategy. To investigate the effects of Peg-IFN-α on the ability of HLA-A*02:01+ patients to respond to this immunotherapeutic tool, PBMCs were stimulated once a week with the pDC line loaded with the HLA-A*02:01-restricted HBc- and HBs-derived peptides, and antigen-specific T cells evaluated using tetramers. Before the start of the treatment (W0), four of five patients tested were unable to respond to the pDC-based stimulation (Fig 5A and 5B) as no amplification of HBc- and HBs-specific T cells were observed upon 14 days of stimulation. Notably, during the course of Peg-IFN-α therapy, the proportion of responder patients increased to 4 of 5 patients (Fig 5B and 5C). Strikingly, this potentiality was maintained for more than 2 years after the cessation of Peg-IFN-α therapy (W144). Thus, Peg-IFN-α treatment potently reversed the unresponsiveness to specific stimulation by HBV-derived peptide-loaded pDCs.

### Correlations between immunologic and clinical parameters

**Fig 6:**
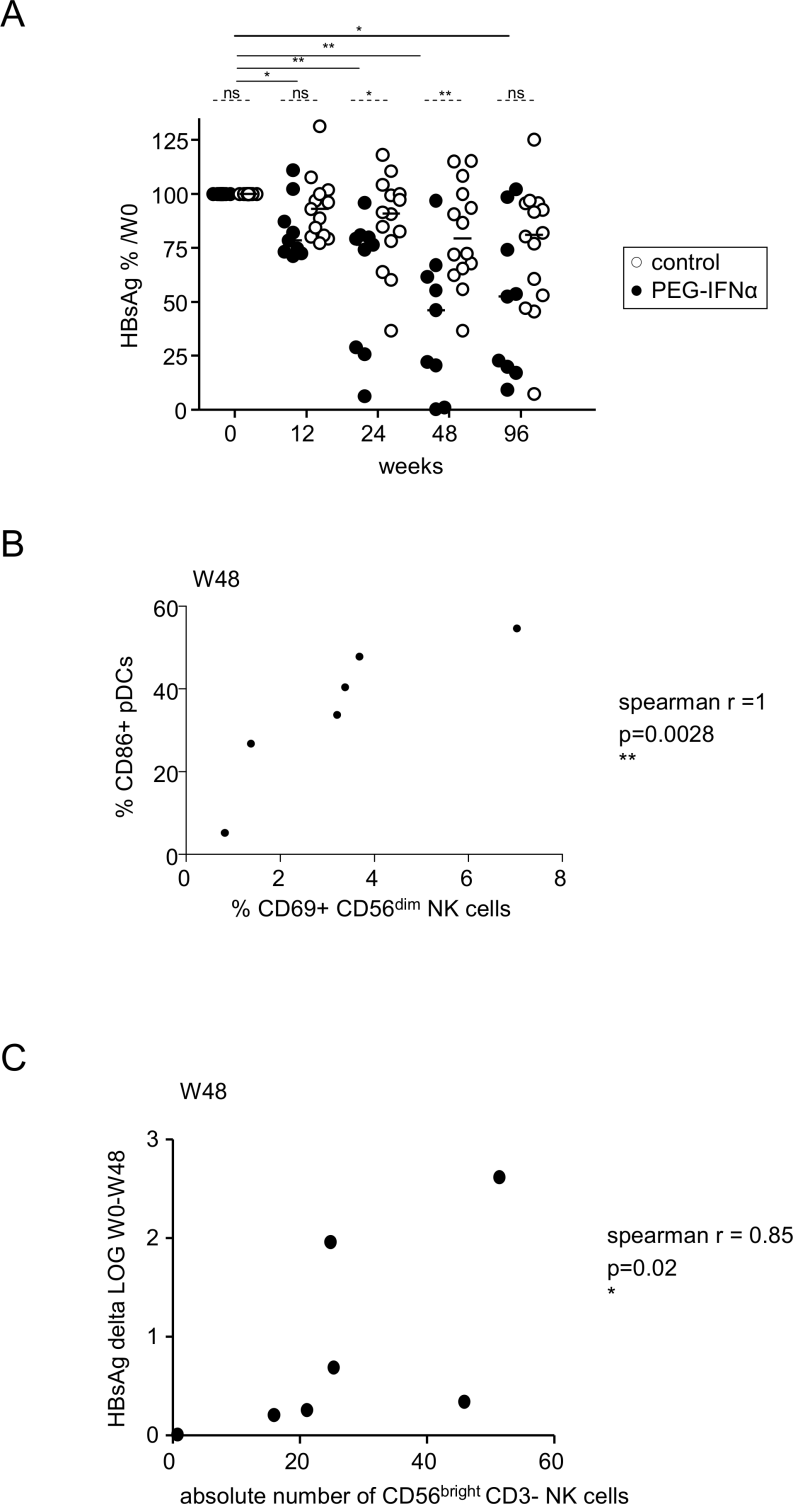
Correlation between immunological parameters and the evolution of HBsAg in patients treated with Peg-IFN-α. (A) Evolution of HBsAg during the course of Peg-IFN-α treatment (percentage of W0). *P*-values were calculated using the Wilcoxon test (*straight lines*) or the Mann-Whitney test (*dashed lines*). Bars represent median. *p<0.05, **p<0.01, ***p<0.001 (B) The modulation of NK cells by Peg-IFN-α correlates with pDC activation. Correlation between the percentage of CD69^+^CD56^dim^ NK cells and the percentage of CD86^+^ pDCs after 48 weeks of treatment (Spearman correlation). (C) The modulation of NK cells by Peg-IFN-α correlates with the decrease in HBsAg. Spearman correlation between the absolute number of CD56^bright^ NK cells and the decline in HBsAg after 48 weeks of treatment.

One of the major signatures of a clinical response to Peg-IFN-α therapy is a decrease in plasma HBsAg levels. In contrast to the control group, the HBsAg level significantly decreased throughout the treatment of patients with the additional Peg-IFN-α therapy compared to baseline (Fig 6A). No correlation between remodeling of the DC/NK/T-cell compartment and the ability to respond to Peg-IFN-α therapy (defined by HBsAg seroconversion at W96) was revealed. However, two patients (#19, #21) underwent a decrease of 2 log IU/ml in HBsAg titer during Peg-IFN-α therapy (Table 1). These patients had the highest levels of both CD86^+^ pDCs and CD69^+^CD56^dim^ NK cells at W48. Interestingly, the proportion of CD86-expressing pDCs strongly correlated with the proportion of CD69-expressing CD56^dim^ NK cells (Fig 6B). Moreover, we observed an inverse correlation between the absolute numbers of CD56^bright^ NK cells and the decline in HBsAg (Fig 6C). In parallel to changes in innate and adaptive immunity, a significant increase in plasma interferon-inducible protein-10 (IP-10) levels was evident throughout the course of the Peg-IFN-α treatment, which may participate in the modulations observed (S7 Fig). Collectively, our data suggest a relationship between the immunologic changes observed and the evolution of viral parameters following Peg-IFN-α therapy.

## Discussion

We provide a dynamic description of the innate and adaptive immunological changes during and up to 2 years after the course of an additional Peg-IFN-α therapy in CHB patients compared to patients receiving NA alone, whose clinical outcomes are known to be different. The findings increase the understanding of Peg-IFN-α immunomodulatory effects, and reveal yet unexplored immune effects of the combination therapy. However, as no seroconversion occurred in our cohort of patients, this work questions which function, direct antiviral or indirect immunomodulation, is more relevant in the efficacy of Peg-IFN-α in the treatment of CHB patients in the add-on settings.

Our data bring a larger view of the immunologic changes triggered by Peg-IFN-α therapy, also with a better subsetting definition and the full kinetics of the immune modulations, reinforcing the results of recent clinical trials [17–20, 23]. Firstly, Peg-IFN-α triggered a marked expansion of the CD56^bright^ NK-cell subset accompanied by an activation and an enhanced functionality of the CD56^dim^ NK-cell subset. CD56^bright^ NK cells represent an intermediate stage of NK-cell differentiation, as a precursor of the CD56^dim^ subset. Their unique immunoregulatory role [25] is illustrated by their preferential interactions with DCs, and their important role in early immune responses and in the shaping of subsequent adaptive responses [26]. Expansion of CD56^bright^ NK cells has been described in several diseases, especially in HCV infection [27], in patients with multiple sclerosis treated with IFN-β [28], and interestingly in patients with active systemic lupus erythematosus [29], a disease mediated by elevated type-I IFN levels. Of note, we observed an inverse correlation between the absolute numbers of CD56^bright^ NK cells and the decline in HBsAg titer. The distinct conclusions on NK functionality compared to other studies [19, 20] may be due to the different ways of data analysis, either as absolute numbers of functional NK cells [19] or as percentages of functional cells within the NK population [20], the time point (point of kinetics or the end of treatment) and the groups that were compared (responders versus non-responders, pre- and on-treatment points).

Moreover, Peg-IFN-α triggered as soon as 4 weeks an activation of pDCs and mDCs, which are critical in antiviral responses [30]. Indeed, type-I IFN is essential for DC maturation and activation [31], and to favor the cross-presentation of antigens by DCs [32], which can help promote subsequent cross-priming of antiviral T cells and facilitate viral clearance. Since DCs are essential to activate NKs and antiviral responses [33], modulation of DCs by Peg-IFN-α may affect the subsequent DC-NK cross-talk and potentiate NK-cell and T-cell functions. This hypothesis is supported by the strong correlation observed between the percentages of activated CD69^+^CD56^dim^ NK cells and CD86^+^ pDCs. Thus, Peg-IFN-α may restore the pDC/NK cross-talk [6], subsequently enhancing antiviral NK-cell responses that could optimize the elimination of infected hepatocytes.

We also revealed key effects of Peg-IFN-α on adaptive immunity. We observed the elicitation of HBc- and HBs-specific CD4 and CD8 immune responses during Peg-IFN-α therapy. This is in contrast with prior observations that Peg-IFN-α did not improve peripheral HBV-specific T-cell responses [17, 22]. The discrepancy may be due to the time of analysis (very early versus late), and/or the use of frozen cells stimulated once with the peptide pools, whereas we performed two rounds of stimulation on freshly isolated PBMCs. Our data are consistent with the previously described restoration of HBV-specific T-cell responses following Peg-IFN-α therapy [23, 34, 35]. In addition, we have shown for the first time the elicitation of Th17 cells upon Peg-IFN-α treatment, preceding the induction of Th1-oriented immunity. Although IL17-producing T cells are described as pro-inflammatory cells and have been associated with liver damage [36], plasma IL17A levels and Th17 cell frequency are negatively correlated with viral load in patients with CHB infection [37]. Such a Th17 profile is revealed only upon specific stimulation with HBV-derived peptide pools and only in the Peg-IFN-α group, suggesting that Th17 cells are either HBV-specific T cells or that HBV-specific T cells subsequently activate other T cells to produce IL17. Interestingly, Th17 cells have been involved in the establishment of long-term immune memory and for promoting B-cell class switch [38]. Thus, they may regulate cellular and humoral antiviral immune responses, therefore favoring the elicitation of HBV-specific immune responses crucial for the immune control of HBV infection.

By potently modulating both the virus fitness and immunity, Peg-IFN-α appears to trigger a systemic immune activation and overcome the functional impairments to subsequently drive antiviral immunity. Despite evident immunologic changes, patients did not achieve HBsAg seroconversion in this cohort. In the overall ANRS HB06 PEGAN trial, HBsAg clearance at W48 has been achieved in only 8% of the patients treated by Peg-IFN-α (7/90) (Bourliere et al. J Hepatol 62, S2, S249, 2015). HBsAg clearance was associated with a low baseline HBs Ag titers and a history of HBeAg seroconversion prior to the inclusion into the trial, which suggests a potential spontaneous anti-viral immune activation. Immune modulations triggered by the therapy might even though improve clinical parameters. The two patients who underwent the highest decline in HBsAg during Peg-IFN-α therapy had the highest level of activated pDCs and NK cells at the end of treatment. The inverse correlation between the absolute numbers of CD56^bright^ NKs and the down-regulation of HBsAg suggested a relationship between the immunologic and virological changes. Strikingly, we identified immunologic changes that persisted for up to 2 years after the cessation of the treatment in the absence of HBsAg seroconversion, as illustrated by the reversion of the unresponsiveness to the peptide-loaded pDCs.

Peg-IFN-α therapy profoundly affects both innate and adaptive immunity, but only transiently and without leading to HBsAg seroconversion. By identifying yet unexplored immune effects of the combination therapy, our study illustrates the pleiotropic action of IFN-α. However, as shown by the other groups, most of the immune changes returned to baseline after the cessation of Peg-IFN-α treatment. The immune alterations induced by Peg-IFN-α treatment failing in the majority of cases to induce seroconversion, they are probably less determinant than the direct antiviral effect of this cytokine. Our work questioned the benefit of the add-on Peg-IFN-α treatment over the NA or Peg-IFN-α monotherapies.

## Supporting Information

S1 Fig Modulation of DC subsets by Peg-IFN-α. Patients with CHB infection were treated with nucleos(t)ide analog alone (*open circles*, n = 11–14) or together with Peg-IFN-α (*black circles*, n = 7–9). Basal percentages of CD40 expression on DC subsets were analyzed by flow cytometry before and at different time points of treatment. The gray area represents the period of Peg-IFN-α administration. Bars represent median. The p-values were calculated using the Wilcoxon test (*straight lines*) or the Mann-Whitney test (*dashed lines*). * p<0.05, ** p<0.01, *** p<0.001.
(TIFF)

S2 Fig Modulation of NK cells subsets by Peg-IFN-α. Patients with CHB infection were treated with nucleos(t)ide analog alone (*open circles*, n = 12–13) or together with Peg-IFN-α (*black circles*, n = 8–9). CD56^+^CD3^-^ total NK cells as well as NK subsets defined based on CD56^bright / dim^ and CD16 expression were analyzed by flow cytometry before and at different time points of treatment. (A) Frequencies of total NK cells, CD56^bright^ NK cells and CD56^dim^ NK cells. (B) Absolute numbers of CD56^bright^CD16^-^ and CD16^+^ NK cells and CD56^dim^CD16^-^ and CD16^+^ NK cells. The gray area represents the period of Peg-IFN-α administration. Bars represent median. The p-values were calculated using the Wilcoxon test (*straight lines*) or the Mann-Whitney test (*dashed lines*). * p<0.05, ** p<0.01, *** p<0.001.
(TIFF)

S3 Fig Basal activation level of NK subsets. The basal level of CD69 expression was analyzed on the NK subsets from patients with CHB infection treated with nucleos(t)ide analog alone (*open circles*, n = 12–13) or together with Peg-IFN-α (*black circles*, n = 8–9) by flow cytometry before and at different time points of treatment. (A) CD69 expression on total NK cells. (B) CD69 expression on CD56^bright^CD16^-^ NK cells and CD56^bright^CD16^+^ NK cells and CD56^dim^CD16^-^ NK cells and CD56^dim^CD16^+^ NK cells. The gray area represents the period of Peg-IFN-α administration. Bars represent median. The p- values were calculated using the Wilcoxon test (*straight lines*) or the Mann-Whitney test (*dashed lines*). * p<0.05, ** p<0.01, *** p<0.001.
(TIFF)

S4 Fig Evaluation of the functionality by NK subsets. Patients with CHB infection were treated with nucleos(t)ide analog alone or together with Peg-IFN-α. (A,B) IFN-γ secretion was evaluated by intracellular staining in total NK cells without or with IL12/IL18 stimulation before and at different time points of treatment. (A) Representative dotplots from patients with CHB infection treated either with nucleos(t)ide analog alone (*upper panel*) or together with Peg-IFN-α (*lower panel*) (gated on total CD56^+^CD3^-^ NK cells). (B) Comparative proportions of IFNγ+ NK cells upon IL12/IL18 stimulation in patients treated with NA alone (*open squares*, n = 12–14) or together with Peg-IFNα (*black squares*, n = 8–9) at different time points of treatment. (C,D) NK cytotoxic activity was evaluated in total NK cells without or with IL12/IL18 stimulation after co-culture with K562 by measuring CD107 surface expression before and at different time points of treatment. (C) Representative dotplots from patients with CHB infection treated either with nucleos(t)ide analog alone (*upper panel*) or together with Peg-IFN-α (*lower panel*) (gated on total CD56^+^CD3^-^ NK cells). (D) Comparative proportions of CD107+ NK cells upon IL12/IL18 stimulation and co-culture with K562 in patients treated with NA alone (*open squares*, n = 12–14) or together with Peg-IFN-α (*black squares*, n = 8–9) at different time points of treatment. For clarity, only the IL12/IL18 condition is shown. The gray area represents the period of Peg-IFN-α administration. Bars represent median. The *p-*values were calculated using the Wilcoxon test (*straight lines*) or the Mann-Whitney test (*dashed lines*). * p<0.05, ** p<0.01, *** p<0.001.
(TIFF)

S5 Fig Basal T-cell features. Patients with CHB infection were treated with nucleos(t)ide analog alone (*open circles*, n = 11–14) or together with Peg-IFN-α (*black circles*, n = 8–9). T-cell subsets were analyzed by flow cytometry before and at different time points of treatment. (A) Evolution of CD4 and CD8 T-cell numbers following Peg-IFN-α treatment. Fold increase in absolute CD4 and CD8 T-cell numbers before and at different time points of treatment. *P*-values were calculated using the Wilcoxon test within the same group of patients toward W0 (*p<0.*05*) and the Mann-Whitney test between the two groups of patients at the indicated time point (#*p<0*.*05)*. (B) Evolution of the basal level of CD69 expression on CD4 T cells (*left panels*) and CD8 T cells (*right panels*). (C) Evolution of the absolute regulatory T-cell (Treg) numbers during the course of treatment. The gray area represents the period of Peg-IFN-α administration. Bars represent median. The p-values were calculated using the Wilcoxon test (*straight lines*) or the Mann-Whitney test (*dashed lines*). * p<0.05, ** p<0.01, *** p<0.001.
(TIFF)

S6 Fig Frequencies of HBV-specific CD4 and CD8 T cells following Peg-IFN-α treatment. The frequencies of HBV-specific CD4/CD8 T cells were evaluated before and during the course of the treatment upon stimulation of PBMC with overlapping peptide pools and intracellular TNFα, IFN-γ and IL-10 cytokine staining from patients treated with nucleos(t)ide analog alone (n = 12–13) or together with Peg-IFN-α (n = 8–9). (A) Frequencies of cytokine-producing HBx- and pol-specific CD4 T-cell responses. (B) Frequencies of cytokine-producing HBx- and pol-specific CD8 T-cell responses.
(TIFF)

S7 Fig Evolution of plasmatic IP-10 levels during the course of Peg-IFN-α treatment. Plasma levels of IP-10 from patients with CHB infection treated with nucleos(t)ide analogue together with Peg-IFN-α (n = 7–9). The gray area represents the period of Peg-IFN-α administration. The p- values were calculated using the Wilcoxon test.
(TIFF)
